# Exosomes Derived from Human Umbilical Cord Mesenchymal Stem Cells Relieve Inflammatory Bowel Disease in Mice

**DOI:** 10.1155/2017/5356760

**Published:** 2017-05-15

**Authors:** Fei Mao, Yunbing Wu, Xudong Tang, Jingjing Kang, Bin Zhang, Yongmin Yan, Hui Qian, Xu Zhang, Wenrong Xu

**Affiliations:** ^1^Key Laboratory of Medical Science and Laboratory Medicine of Jiangsu Province, School of Medicine, Jiangsu University, Zhenjiang, Jiangsu 212013, China; ^2^Jiangsu University of Science and Technology, Zhenjiang, Jiangsu 212018, China

## Abstract

Exosomes secreted by mesenchymal stem cells (MSCs) have shown repairing effects on several tissue injury diseases. In this study, we aimed to investigate the effects of exosomes released from human umbilical cord mesenchymal stem cells (hucMSCs) on the treatment of dextran sulfate sodium- (DSS-) induced inflammatory bowel disease (IBD) and to explore the underlying mechanism. We found that indocyanine green (ICG) labeled exosomes homed to colon tissues of IBD mice at 12 hours after injection. Exosomes significantly relieved the severity of IBD in mice as hucMSCs. The expression of IL-10 gene was increased while that of TNF-*α*, IL-1*β*, IL-6, iNOS, and IL-7 genes was decreased in the colon tissues and spleens of exosomes-treated mice. Furthermore, the infiltration of macrophages into the colon tissues was decreased by exosome treatment in IBD mice. In addition, we provided evidence that in vitro coculture with exosomes inhibited the expression of iNOS and IL-7 in mouse enterocoelia macrophages. Moreover, we found that the expression of IL-7 was higher in the colon tissues of colitis patients than that of healthy controls. Our findings suggest that exosomes from hucMSCs have profound effects on alleviating DSS-induced IBD and may exert their impact through the modulation of IL-7 expression in macrophages.

## 1. Introduction

Inflammatory bowel disease (IBD) is a noninfectious, chronic, systemic, and relapsing inflammatory disorder of the gastrointestinal tract. IBD is characterized by recurrent and long-lasting episodes of diarrhea and abdominal pain, which primarily includes ulcerative colitis (UC) and Crohn's disease (CD) [1]. The incidence of IBD increases rapidly in the western countries [2, 3]. For example, IBD is estimated to affect 1–1.5 million of people in USA [4]. IBD patients have reduced life quality and increased mortality risk because of colitis-associated colorectal cancer (CAC), which accounts for an estimated 15% of deaths in IBD patients [5–7]. Thus, the intervention of IBD would decrease the incidence of gastrointestinal cancers. Currently, IBD treatment mainly depends on anti-inflammatory drugs, immune-modifying agents or immunomodulators, thiopurine agents, and anti-TNF monoclonal antibodies; however, the efficacy of IBD treatment is still unsatisfactory [8]. Therefore, there is an urgent need to seek for new approaches for IBD treatment.

Cell therapies have shown promising effects on the treatment of IBD [9]. Mesenchymal stem cells (MSCs) are defined as cells that can self-renew and differentiate into various mesoderm lineage cell types, such as adipocytes, myocardiocytes, chondrocytes, and osteoblasts [9–12]. Although MSCs can be found in most adult tissues, the major sources of MSCs for therapeutic use are bone marrow, umbilical cord, and adipose tissues [13]. The previous studies have shown that MSCs transplantation exerts promising effects on the treatment of many diseases through their immunosuppressive functions [14–16]. MSCs have been used to treat tissue injury diseases such as myocardial infarction [17], acute renal failure [18], acute liver injury [19], and collagen-induced arthritis (CIA) [16].

Exosomes are nanosized extracellular vesicles secreted by cells and have been described as a new mechanism for cell-to-cell communication [20]. In recent years, a number of studies have shown that exosomes secreted by MSCs also have the ability to serve as a therapeutic tool in the repair of tissue injuries including liver fibrosis [21], renal failure [22], acute tubular injury [23, 24], and myocardial ischemia/reperfusion [25]. We have previously reported the potential of exosomes from human umbilical cord MSCs in the treatment of liver and kidney injuries [21, 22]. Whether exosomes from hucMSCs can be used in the treatment of DSS-induced IBD remains unknown. In this study, we demonstrated that exosomes derived from hucMSCs could alleviate DSS-induced IBD in mice, which may provide a new approach for the treatment of IBD.

## 2. Materials and Methods

### 2.1. Cell Culture and Exosomes Extraction

MSCs were isolated from human umbilical cord and cultured as previously described [26]. HucMSCs were cultured in complete DMEM (low glucose, Invitrogen, USA) containing 10% fetal calf serum (Invitrogen, USA) at 37°C in humid air with 5% CO_2_. For exosome extraction, hucMSCs at passage 4 were cultured in serum-free medium for 48 h. The conditioned medium was collected and centrifuged at 1,000*g* for 20 minutes to remove cell debris, followed by centrifugation at 2,000*g* for 20 minutes and 10,000*g* for 20 minutes. The supernatant was collected and concentrated using 100 KDa MWCO (Millipore, USA) at 1,000*g* for 30 minutes. The concentrated supernatant was loaded upon 5 ml of 30% sucrose/D_2_O cushions and then ultracentrifuged at 100,000*g* for 60 minutes (optimal-90K, Beckman Coulter). The microvesicles-enriched fraction was harvested and diluted with PBS and then centrifuged thrice at 1,000*g* for 30 minutes using 100 KDa MWCO. Finally, the purified exosomes were collected and subjected to filtration on 0.22 *μ*m pore filter (Millipore, USA) and stored at −70°C for future use [27].

Macrophages were obtained from the peritoneal cavity of normal eight-week-old male KM mice according to the previous report [28]. The cells were harvested in cold RPMI 1640 medium (Gibco, Thermo Scientific, USA) containing heparin (1 : 20,000) and were maintained in RPMI 1640 medium containing 10% fetal calf serum. Macrophages (10^6^ cells/well) were added to 6-well plate (Corning, Life Sciences, USA) and incubated at 37°C in humid air with 5% CO_2_. Macrophages were incubated with hucMSCs derived exosomes (160 *μ*g/ml) [29] for 72 h and harvested for further studies.

### 2.2. Characterization of hucMSC Exosomes

20 *μ*l drops of purified exosomes were adsorbed onto copper grids, placed for 1 minute at room temperature, adsorbed onto the superfluous exosomes, and stained with 30 g/L phosphotungstic acid (pH 6.8) for 5 minutes at room temperature, and the sample dried under half-watt lamp. Samples were imaged using a transmission electron microscopy (FEI Tecnai 12, Philips) [27]. The protein content of hucMSC exosomes was tested by a BCA Protein Assay kit (CWbio), and the number of hucMSC exosomes was quantified using nanoparticle tracking analysis, as described previously [30]. The CD9 (Bioworld Technology, USA), CD63 (Bioworld Technology, USA), and CD81 (Epitomics, USA) molecules, which were frequently located on the surface of exosomes, were analyzed using western blotting [31].

### 2.3. Animal Model

Male KM mice (Laboratory Animal Research Center of Jiangsu University, Jiangsu, China) aged 7 weeks were randomly divided into 4 groups (*n* = 6/group): control group (normal), IBD group (IBD), exosomes-treated IBD group (Ex + IBD), and hucMSCs-treated IBD group (hucMSC + IBD). All experimental procedures were conducted in accordance with the Animal Use and Care Committee of Jiangsu University. For the IBD model, mice were exposed to 3% DSS (MP, Cat NO: 160110, Canada) in the drinking water for 11 days. On days 3, 6, and 9, the mice in Ex + IBD group were injected with 400 *μ*g exosomes/mouse [30], and the mice in hucMSC + IBD group were injected with 1.3 × 10^6^ hucMSCs/mouse which could release about 400 *μ*g exosomes. The mice in IBD group were injected with PBS through the tail vein. Mice were weighed and the mouse stool was monitored daily. All the mice were sacrificed at day 11. The colon tissues and spleens were collected for further studies.

For in vivo exosomes trafficking assay, 400 *μ*g exosomes were incubated with 10 *μ*g/ml indocyanine green (ICG) (Ouhe Technology, Beijing, China). At 12 hours after incubation, the labeled exosomes were collected and injected into the mice through the tail vein. The presence of labeled exosomes in colon tissues and spleens was detected in a live animal imaging system.

### 2.4. Total RNA Extraction and qRT-PCR

The RNA was extracted from the colon mucosa and splenic mononuclear cells using TRIzol Reagent (Life technologies, Carlsbad, CA, USA). Equal amount of RNA was used for real-time RT-PCR analyses. The cDNAs were synthesized by using the HiScript 1st Strand cDNA Synthesis Kit (Vazyme Biotech, Shanghai, China). The gene expression was analyzed in a Step One Plus Real-Time PCR System (Applied Biosystems, Life Technologies, USA) as previously described [32]. *β*-Actin was used as an internal control. The sequences of specific primers are listed in Table 1.

### 2.5. Western Blotting Analysis

The colon mucosa and splenic mononuclear cells were homogenized in modified RIPA lysis buffer supplemented with proteinase inhibitors (Vazyme biotech, Shanghai, China). Fifty micrograms of protein samples was separated on a 10% SDS-PAGE (sodium dodecyl sulfate-polyacrylamide gel electrophoresis). After electrophoresis, the proteins were transferred to PVDF (polyvinylidene difluoride) membrane (Millipore, IPVH00010, USA). The membrane was blocked in 5% nonfat milk for 1 h at room temperature and incubated with the corresponding primary antibodies at 4°C overnight. The sources of the antibodies were anti-IL-7 (Santa Cruz Biotechnology, USA), anti-CD9 (Bioworld Technology, USA), anti-CD63 (Bioworld Technology, USA), anti-CD81 (Epitomics, USA), and anti-*β*-actin (Santa Cruz Biotechnology). The next day, the blots were incubated with the secondary antibodies for 1 h at room temperature. The blots were visualized by chemiluminescence (Millipore, USA) and detected by using the imaging software (GE Healthcare, Life Sciences, USA).

### 2.6. Immunohistochemistry (IHC) Staining

Formalin-fixed paraffin-embedded colon tissues and spleens of mouse and colon tissues of normal control and colitis patients were sectioned (4 *μ*m thick), mounted on slides, and stained by using hematoxylin-eosin (HE). All clinical procedures followed the protocols approved by the ethical committee of the ethical committee of Jiangsu University and the methods were carried out in accordance with the approved guidelines. For the detection of proliferating cell nuclear antigen (PCNA), CD206, and IL-7, the tissue slides were incubated with the primary antibodies against PCNA (1 : 100, Bioworld Technology, USA), CD206 (1 : 100, Santa Cruz Biotechnology), and IL-7 (1 : 100, Santa Cruz Biotechnology) followed by biotinylated sheep anti-rabbit IgG (Bostar, Wuhan, China). The signal was developed by staining with DAB (3,3′-diaminobenzidine), and the nuclei were slightly counterstained with hematoxylin for microscopic examination.

### 2.7. Statistical Analysis

The experimental values were expressed as mean ± standard deviations (SD). The significant differences between two groups were analyzed by ANOVA for unpaired *t*-test followed by the Holm-Bonferroni. At least three independent experiments were performed to confirm the reproducibility of each experiment. The significant difference was evaluated by Prism software (Graph Pad, San Diego, USA), and *P* < 0.05 was considered statistically significant.

## 3. Results

### 3.1. Characterization of hucMSC Exosomes

**Figure 1:**
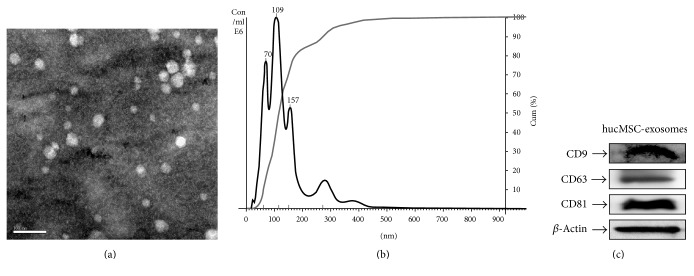
Characterization of hucMSC derived exosomes. (a) Transmission electron microscopy analysis of exosomes secreted by hucMSCs. Scale bar: 100 nm. (b) Nanoparticle tracking analysis of exosomes. (c) CD9, CD63, and CD81 expressions in exosomes were detected by western blotting.

Transmission electron microscopy analysis showed spheroid morphology of the purified exosomes, with a mean diameter of 40–100 nm (Figure 1(a)). The particle pictorial diagram of exosomes and particle size distribution were recorded by nanoparticle tracking analysis (Figure 1(b)). The purified exosomes expressed CD9, CD63, and CD81 (Figure 1(c)). In conclusion, these results indicate that we have successfully isolated and identified exosomes from hucMSCs.

### 3.2. Exosomes Home to the Colon Tissues and Spleens of the IBD Mice

**Figure 2:**
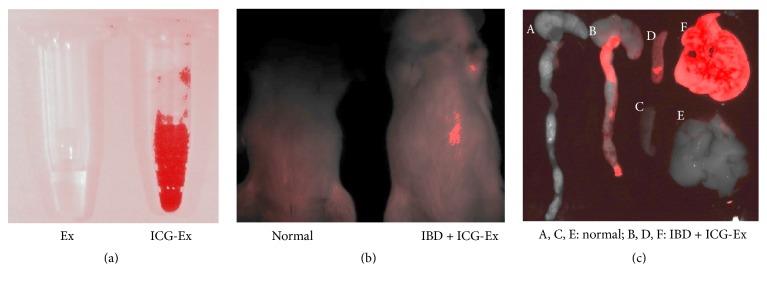
Live animal imaging analyses for the homing of ICG-exosomes to colon tissue and spleen of IBD mice. (a) Representative images of exosomes labeled with ICG for 12 hours were detected under live animal imaging system. (b) The IBD mice received intravenous injection of ICG-exosomes for 12 hours and were detected under live animal imaging system (*n* = 3). (c) The colon tissues, spleens, and livers of IBD mice treated with ICG-exosomes were detected under live animal imaging system.

According to the previous study [2], we first established the DSS-induced IBD mouse model. To study the homing of exosomes to the injured colon tissues of IBD mice, we labeled exosomes with ICG (Figure 2(a)) and injected the labeled exosomes into IBD mice through the tail vein. At 12 hours after injection, the mice were anaesthetized and examined using the live animal imaging system. The results showed the IBD mice injected with ICG-exosomes showed red fluorescence at the abdomen (Figure 2(b)). The mice were sacrificed, and the colon tissues, spleens, and livers were examined using the live animal imaging system. The colon tissues, spleens, and livers of IBD mouse injected with ICG-exosomes showed a strong red fluorescence (Figure 2(c)). On the contrary, the colon tissues, spleens, and livers of normal mice had no red fluorescence. These results indicate that the injected exosomes could home to the colon tissues, spleens, and livers of the IBD mice.

### 3.3. Exosomes from hucMSCs Attenuate the Severity of DSS-Induced IBD in Mice

**Figure 3:**
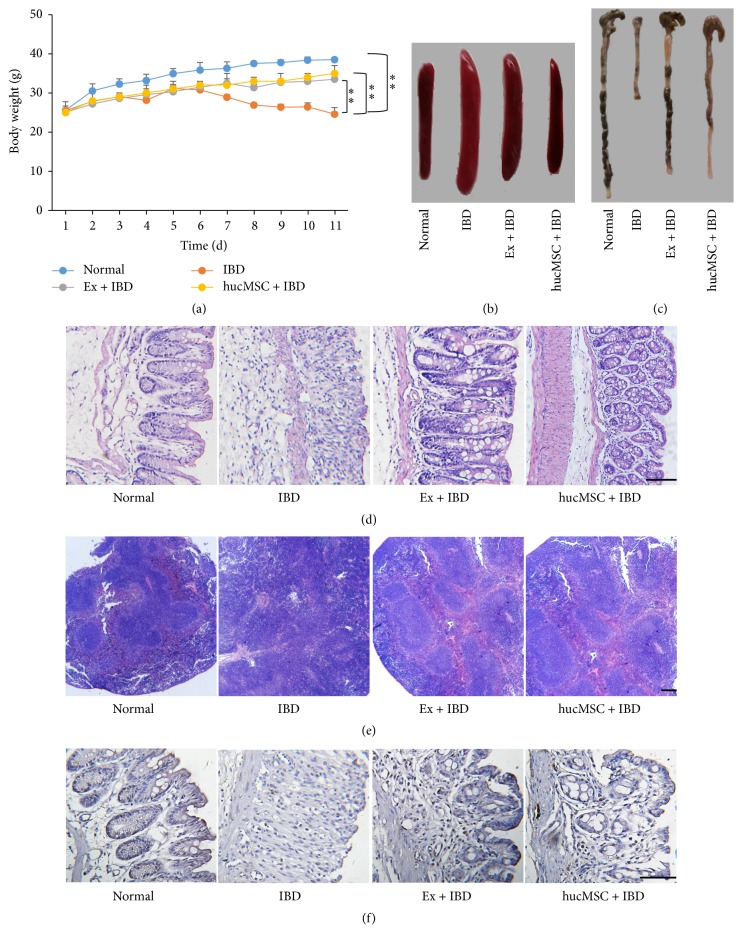
The effects of hucMSC derived exosomes on DSS-induced IBD in mice. (a) The body weight of mice in normal, IBD, Ex + IBD, and hucMSC + IBD groups. (b) The size of the spleens of mice in normal, IBD, Ex + IBD, and hucMSC + IBD groups. (c) The colon lengths of mice in normal, IBD, Ex + IBD, and hucMSC + IBD groups. (d) HE staining of the colon tissues of mice in normal, IBD, Ex + IBD, and hucMSC + IBD groups. (e) HE staining of the spleens of mice in normal, IBD, Ex + IBD, and hucMSC + IBD groups. (f) Representative photographs of immunohistochemical staining of PCNA in the colon tissues of mice in normal, IBD, Ex + IBD, and hucMSC + IBD groups (*n* = 6). Data shown were representative of three independent experiments. Bars represent the means ± SD. Scale bar = 100 *μ*m. ^*∗∗*^*P* < 0.01.

Based on the established model, we investigated the effects of exosomes from hucMSCs on DSS-induced IBD. The results showed that IBD mice had blood stool at 5 days after exposure to DSS and began to lose weight at 7 days after DSS exposure. As that were observed for hucMSCs, the treatment with exosomes from hucMSCs significantly inhibited weight loss in IBD mice (Figure 3(a)), indicating that exosomes could alleviate the development of IBD. The size of spleen in exosomes-treated group was significantly smaller than that in IBD group (Figure 3(b)). The mouse colon length in exosomes-treated group was longer than that in IBD group (Figure 3(c)). IBD mice showed destroyed structure integrity of colon tissues accompanied with increased inflammatory cell infiltration. In contrast, exosomes treatment recovered the structure integrity of colon tissues and reduced the infiltration of inflammatory cells (Figure 3(d)). Furthermore, the splenic nodules were broken in IBD mice but were much more integral in exosomes-treated mice (Figure 3(e)). The immunohistochemical staining results of PCNA indicated that the percentage of proliferating cells was decreased in the colon tissues of IBD mice while increased in that of exosomes-treated mice, suggesting that the proliferating ability of colon mucosa epithelial cells was recovered by exosomes treatment (Figure 3(f)).

### 3.4. Exosomes from hucMSCs Regulate the Expression of Cytokines in the Colon Tissues and Spleens of IBD Mice

**Figure 4:**
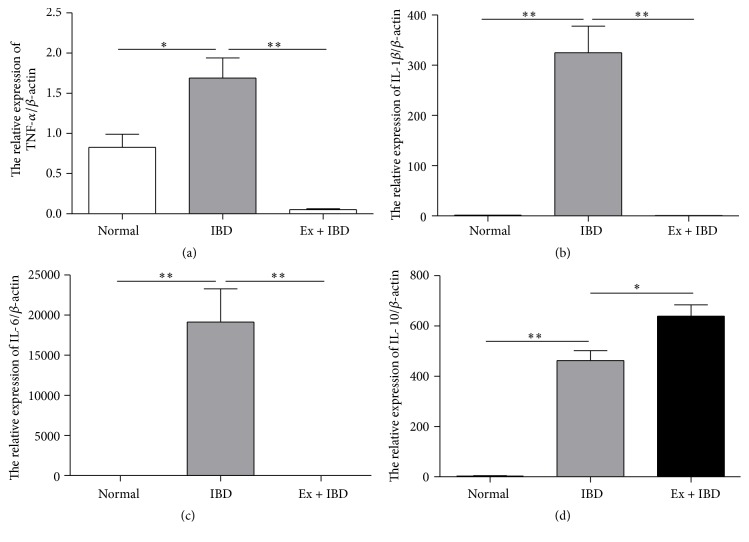
QRT-PCR analyses of the gene expression levels of inflammation-associated cytokines in the colon tissues of mice in normal, IBD, and Ex + IBD groups. (a) TNF-*α*, (b) IL-1*β*, (c) IL-6, and (d) IL-10. Data shown were representative of three independent experiments. Bars represent the means ± SD. ^*∗*^*P* < 0.05; ^*∗∗*^*P* < 0.01.

**Figure 5:**
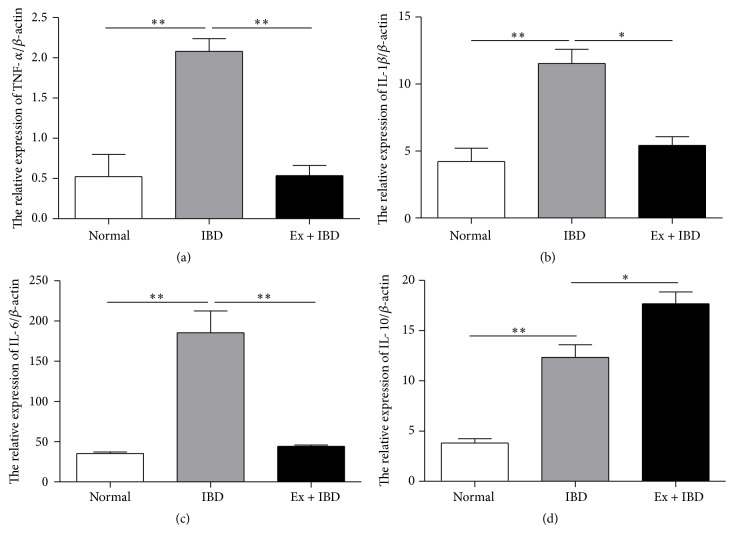
QRT-PCR analyses of the gene expression levels of inflammation-associated cytokines in the spleens of mice in normal, IBD, and Ex + IBD groups. (a) TNF-*α*, (b) IL-1*β*, (c) IL-6, and (d) IL-10. Data shown were representative of three independent experiments. Bars represent the means ± SD. ^*∗*^*P* < 0.05; ^*∗∗*^*P* < 0.01.

We next wanted to know whether exosomes attenuate DSS-induced IBD by regulating the inflammatory responses. Thereafter, we investigated the expression of several proinflammatory cytokines in the colon tissues and splenic mononuclear cells of IBD mice. The results of qRT-PCR showed that the expression of proinflammatory cytokines such as TNF-*α*, IL-1*β*, and IL-6 increased in the colon tissues of IBD mice while decreased in that of exosomes-treated mice (Figures 4(a)–4(c)). In contrast, the expression of anti-inflammatory cytokine IL-10 was increased in the colon tissues of exosomes-treated IBD mice compared to that in IBD mice (Figure 4(d)). The similar results were found in the splenocytes from the IBD mice treated with exosomes (Figure 5).

### 3.5. Exosomes from hucMSCs Inhibit IL-7 Expression in the Colon Tissues and Spleens of IBD Mice

**Figure 6:**
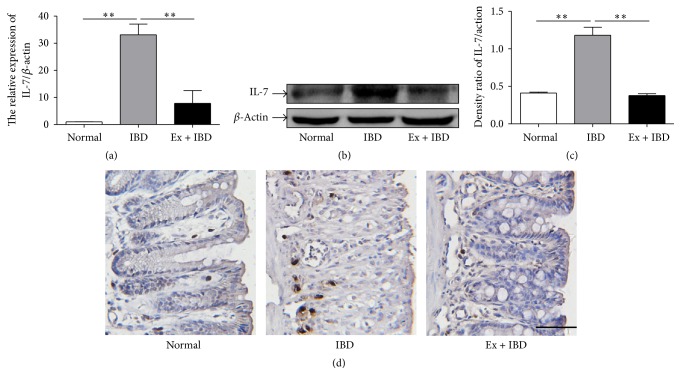
The expression levels of IL-7 in the colon tissues of mice in normal, IBD, and Ex + IBD groups. (a) The expression of IL-7 was measured by using qRT-PCR. (b) The protein levels of IL-7 were measured by using western blot. (c) Densitometric analyses of the protein bands in (b). (d) Representative photographs of the immunohistochemical staining of IL-7. Data shown were representative of three independent experiments. Bars represent the means ± SD. Scale bar = 100 *μ*m. ^*∗∗*^*P* < 0.01.

**Figure 7:**
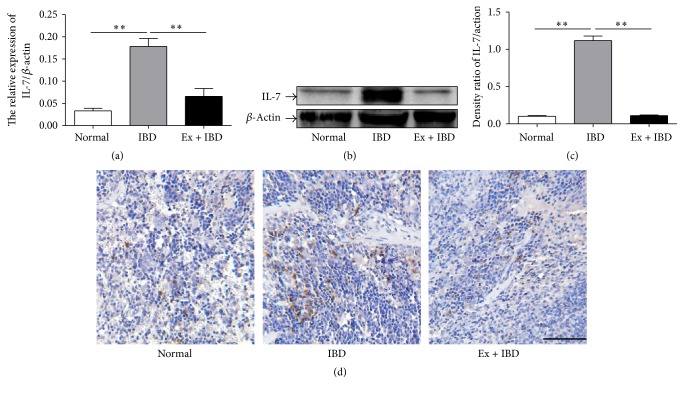
The expression levels of IL-7 in the spleens of mice in normal, IBD, and Ex + IBD groups. (a) The expression of IL-7 was measured by using qRT-PCR. (b) The protein levels of IL-7 were measured by using western blot. (c) Densitometric analyses of the protein bands in (b). (d) Representative photographs of the immunohistochemical staining of IL-7. Data shown were representative of three independent experiments. Bars represent the means ± SD. Scale bar = 100 *μ*m. ^*∗∗*^*P* < 0.01.

IL-7 has been reported to play an important role in the development of IBD [33]. We then detected the expression of IL-7 in the colon tissues and the spleens of IBD mice. The expression of IL-7 gene and protein was significantly increased in the colon tissues of IBD mice but decreased in that of exosomes-treated IBD mice (Figures 6(a)–6(c)). The results of immunohistochemical staining for IL-7 in the colon tissues indicated that the number of IL-7+ cells increased in the colon tissues of IBD mice while exosomes treatment significantly reduced the number of IL-7+ cells (Figure 6(d)). We further investigated the expression of IL-7 in the spleens of IBD mice. The results were similar to that observed in the colon tissues (Figure 7).

### 3.6. Exosomes from hucMSCs Inhibit the Infiltration of Macrophages in IBD Mice

**Figure 8:**
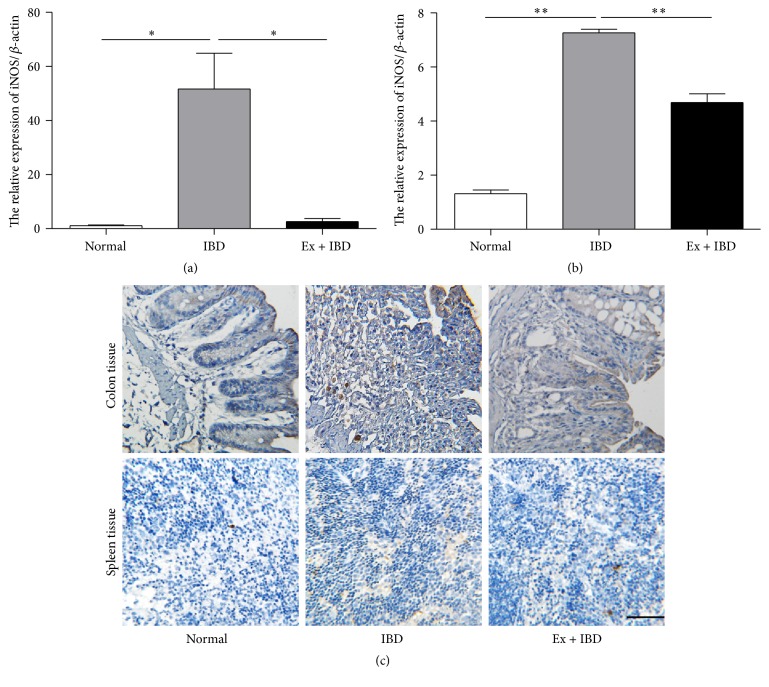
The expression of iNOS and CD206 in the colon tissues and spleens of mice in normal, IBD, and Ex + IBD groups. (a) The expression of IL-7 was measured by using qRT-PCR in the colon tissues of mice in normal, IBD, and Ex + IBD groups. (b) The expression of IL-7 was measured by using qRT-PCR in the spleens of mice in normal, IBD, and Ex + IBD groups. (c) Representative images of the immunohistochemical staining of CD206 in the colon tissues and spleens of mice in normal, IBD, and Ex + IBD groups. Data shown were representative of three independent experiments. Bars represent the means ± SD. Scale bar = 100 *μ*m. ^*∗*^*P* < 0.05; ^*∗∗*^*P* < 0.01.

Macrophages are critical cells involved in IBD and they have been suggested as one of the major sources of IL-7. Therefore, we detected the expression of iNOS and the infiltration of macrophages in the colon tissues. The results of qRT-PCR showed that the expression of iNOS increased significantly in IBD mice while decreased in exosomes-treated mice (Figures 8(a)-8(b)). The results of immunohistochemical staining showed that there was an increased infiltration of CD206+ macrophages in IBD mice. On the contrary, exosomes treatment decreased the number of CD206+ macrophages in IBD mice (Figure 8(c)).

### 3.7. Exosomes from hucMSCs Inhibit IL-7 Expression in Macrophages

**Figure 9:**
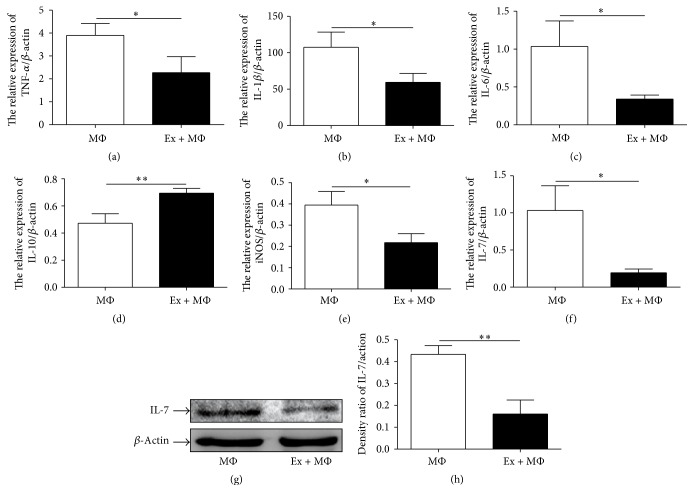
The expression of IL-7 and inflammation-associated cytokines in macrophages cocultured with hucMSCs derived exosomes. (a) TNF-*α*, (b) IL-1*β*, (c) IL-6, (d) IL-10, (e) iNOS, and (f) IL-7. (g) The protein levels of IL-7 were measured by using western blot. (h) Densitometric analyses of the protein bands in (g). Data shown were representative of three independent experiments. Bars represent the means ± SD. ^*∗*^*P* < 0.05; ^*∗∗*^*P* < 0.01.

We isolated macrophages from the peritoneal cavity of normal mouse and cocultivated them with exosomes from hucMSCs. Consistent with that observed in vivo, exosomes decreased the expression of TNF-*α*, IL-1*β*, IL-6, iNOS, and IL-7 but increased the expression of IL-10 in macrophages in vitro (Figure 9).

### 3.8. IL-7 Expression Is Increased in Colitis Patients

**Figure 10:**
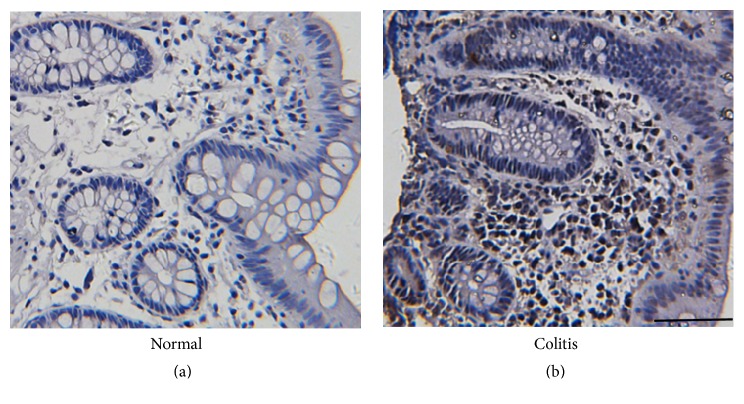
Representative images of the immunohistochemical staining of IL-7 in the colon tissues from colitis patients and normal control. Scale bar = 100 *μ*m.

Furthermore, we detected the expression of IL-7 in the colon tissues of normal controls and colitis patients by using immunohistochemistry. We found that there was more IL-7+ cells in the colon tissues of colitis patients than that in normal controls (Figure 10).

## 4. Discussion

The therapeutic effects of MSCs on IBD and other diseases have previously been reported [9, 16–19], but the exact mechanism is unclear. Some studies have shown that donor MSCs exhibit the effects by releasing paracrine factors and their engraftment to the targeted site is not required [34, 35]. Exosomes, formed by fusion of multivesicular endosomes with the plasma membrane and released by most cell types, are considered to play an important role in transmitting information between cells [36]. In addition to soluble factors, exosomes have also been proposed as a new mechanism underlying the paracrine action of MSCs. The work from our group and the others have shown that exosomes from MSCs have therapeutic effects on several tissue injury diseases such as liver fibrosis, cisplatin-induced renal oxidative stress, ischemia/reperfusion-induced acute and chronic kidney injury, and myocardial ischemia/reperfusion injury [21–25]. In this study, we further demonstrated that exosomes released by hucMSCs could ameliorate DSS-induced IBD in mice.

IBD is a chronic and progressive inflammatory state of the gastrointestinal tract and the colon mucosal lesion is characterized by the infiltration of inflammatory cells, which mainly include macrophages. Macrophages can be stimulated to secrete different kinds of cytokines and enzymes, which would result in the injury of intestine tissue [37]. We demonstrated that the expression of iNOS and the recruitment of macrophages to the inflamed tissues were reduced in exosomes-treated IBD mice. In addition, the treatment with exosomes from hucMSCs decreased the expression of proinflammatory cytokines such as TNF-a, IL-1*β*, and IL-6 and increased the expression of anti-inflammatory cytokine IL-10. Our findings are in accordance with those reported in a previous study showing that the repression of monocyte recruitment and macrophage polarization alleviates colitis [38]. Shen et al. reported that CCR2+ exosomes released by MSCs suppressed the functions of macrophages and alleviated ischemia/reperfusion-induced renal injury [39]. The results of Li et al. indicated that hucMSC exosomes suppressed LPS-induced macrophage inflammation and attenuated burn-induced excessive inflammation [40]. In brief, exosomes released by MSCs might alleviate tissue injury by inhibiting the functions of macrophages.

IL-7 is a pleiotropic cytokine that acts as the mitogen, growth, and survival factor for the growth and homeostasis of T lymphocytes [41]. IL-7 is proved to be an important cytokine that activates mucosal inflammation in IBD. The downregulation of IL-7 in mice with DSS-induced colitis could inhibit inflammation in the gastrointestinal tract [42]. Nemoto et al. suggest that IL-7 is an essential factor for the persistence of chronic T-cell-mediated colitis, which is constitutively produced by intestinal goblet cells [33]. The previous studies demonstrate that the serum level of IL-7 is increased in IBD patients [43, 44]. In this study, we found that exosomes from hucMSCs significantly inhibited the expression of IL-7 in the colon mucosa tissues and spleens of IBD mice. We further demonstrated that exosomes from hucMSCs inhibited the expression of IL-7 in peritoneal macrophages, which suggests that exosomes treatment may have a direct suppressive effect on IL-7 expression in macrophages. We also found that IL-7 expression increased in the colon tissues of colitis patients than that in normal controls, which was similar to the previous studies [43, 44].

The composition of exosomes is variable and cell origin-specific. Wong et al. performed a proteomic analysis of serum exosomes from DSS-induced colitis mouse and identified 56 differentially expressed proteins in exosomes, a majority of which were acute-phase proteins and immunoglobulins. The results of bioinformatics analysis suggested that these proteins were mainly involved in the complement and coagulation cascade, which has been implicated in macrophage activation [45]. A recent study pointed out that human bone marrow MSC-derived extracellular vesicles (BMSC-EVs) had 365 known mature miRNAs [46]. However, which kind of specific miRNA in BMSC-EVs contributed to their biological activities was not clear. In our previous study, we found that hucMSC-Ex derived Wnt4 was critical for its repairing effect on cutaneous wound healing [47]. We also demonstrated that hucMSC-Ex-derived glutathione peroxidase 1 (GPX1) promotes the recovery of hepatic oxidant injury [48]. Lai et al. identified 857 proteins inside exosomes released by MSCs-derived embryonic stem cell lines [49]. Kim et al. characterized the protein composition of bone marrow MSC-derived microvesicles (MVs) and identified 730 proteins, including several self-renewal and differentiation mediators [50]. We hypothesize that the protein contents in hucMSC exosomes may play a key role in tissue injury repair. However, a proteomic analysis of hucMSC exosomes is needed to help reveal the mechanism responsible for the role of hucMSC exosomes in attenuating DSS-induced IDD in mice.

In conclusion, our findings suggest that exosomes from hucMSCs inhibit the expression of IL-7 in macrophages and relieves inflammatory responses, which attenuates DSS-induced colitis in mice. The use of hucMSCs derived exosomes may provide a novel approach for the treatment of IBD.

## Figures and Tables

**Table 1:** Primer sequences for RT-PCR.

Primer sequences for the amplification of target genes and *β*-actin			
Genes	Primer sequence (5′-3′)	Annealing temp. (°C)	Amplicon size (bp)
IL-7	FOR: CGCAGACCATGTTCCATGTT	60	268
	REV: AACTTGCGAGCAGCACGATT		
TNF-*α*	FOR: AACTCCAGGCGGTGCCTATG	63	242
	REV: TCCAGCTGCTCCTCCACTTG		
IL-1*β*	FOR: AGCTTCAGGCAGGCAGTATC	61	215
	REV: TCATCTCGGAGCCTGTAGTG		
IL-10	FOR: CCTGGCTCAGCACTGCTATG	61	151
	REV: TCACCTGGCTGAAGGCAGTC		
IL-6	FOR: AAGTCCGGAGAGGAGACTTC	58	487
	REV: TGGATGGTCTTGGTCCTTAG		
iNOS	FOR: CAGCTGGGCTGTACAAACCTT	61	95
	REV: CATTGGAAGTGAAGCGTTTCG		
